# The Ubiquitin–26S Proteasome System—A Versatile Player Worthy of Close Attention in Plants

**DOI:** 10.3390/ijms24098185

**Published:** 2023-05-03

**Authors:** Zhihua Hua

**Affiliations:** 1 Department of Environmental and Plant Biology, Ohio University, Athens, OH 45701, USA; hua@ohio.edu; 2 Interdisciplinary Program in Molecular and Cellular Biology, Ohio University, Athens, OH 45701, USA

In the crowded and confined space of a cell, numerous proteins work collaboratively in various subsystems, such as metabolic pathways, organelle compartments, and complexes, to regulate cell growth and development. Given the estimated concentration range from 20% to 30% (w/v) in terms of the total cellular protein [1], the average size of 52 kDa ascribed to human proteins [2,3], and the median 50 μm in diameter of human cells [4], it can be estimated that there are about 1.5 ~ 2.3 × 10^11^ protein molecules in one human cell. It has also been estimated that plants, such as *Arabidopsis thaliana*, have an average protein size and cellular volume similar to those of humans [4,5]. Thus, there are myriad proteins in both human and plant cells. How such a large cohort of proteins is organized and managed for keeping intracellular proteome homeostasis constitutes the central topic in molecular and cellular biology.

One necessary mechanism is the timely removal of any outdated and/or abnormal proteins, which is primarily executed by the ubiquitin (Ub)–26S proteasome system (UPS) [6]. Recently, selective autophagy pathways have also emerged as the second important system for degrading large protein complexes or aggregates in addition to damaged organelles [7]. The essential role of the UPS in safeguarding intracellular proteome homeostasis is reflected by its dramatic expansion in eukaryotic organisms, particularly in plants. For example, using a closing-target-trimming in-depth annotation algorithm [8,9], we discovered 1460 loci (5.3% of all protein-coding genes) in the Arabidopsis genome that encode a UPS member [10]. The sheer size of the UPS strongly suggests that this post-translational modification (PTM) rivals gene transcription in both depth and breadth as a dominant regulatory mechanism controlling plant growth and development, which can be partly gauged in the collection of studies included in this Special Issue of the *International Journal of Molecular Sciences*. In this collection of eight articles, the involvement of the UPS is identified in areas as diverse as hormone signaling [11,12,13], stress response [11,12,13], pathogen defense [14], chloroplast function [15], floral development [13,16], plasma membrane protein sorting [17], and crosstalk with other major gene expression regulatory machineries such as microRNA-mediated post-transcriptional regulation [13], histone epigenetic modifications [16], and autophagy [18].

According to the composition of the UPS and its wide range of regulatory pathways, it can be studied by either characterizing the functions of its individual members or identifying its members involved in a particular growth and developmental process.

The UPS is composed of two subsystems: the ubiquitylation pathway and the 26S proteasome. The former involves a conserved E1 ubiquitin-activating enzyme, multiple E2 ubiquitin-conjugating enzymes, and a remarkable number of E3 ubiquitin ligases [19,20], while the latter comprises a minimum of 34 subunits that are encoded by 53 loci in Arabidopsis [18,21]. Many studies in plant biology have utilized a reverse genetics approach to characterize the functions of UPS members in different subfamilies. In this issue, four articles studied the functions of plant U-box E3 ligases [13], Really Interesting New Gene (RING) Domain Ligase (RGLG)-type E3 ligases (RGLGs; [17]), the 26S proteasome [18], and a specific deubiquitylation enzyme (DUB) called Ovarian Tumor (OTU) 5 [16].

In the research conducted by Mao et al. [13], the authors provided a comprehensive review of the present understanding of the plant U-box (PUB) E3 ligases with respect to several areas, including classification and evolutionary characterization, gene expression regulation, and multiple biological functions. Similar to a RING-finger domain, a U-box domain is also defined by a stretch of ~70 amino acids that form a β-β-α-β-α fold [22]. However, unlike the RING-finger domain, the U-box does not possess zinc-chelating activity, rendering U-box domain-containing E3 ligases a separated family from the RING-finger-domain-containing mono-subunit E3 family. In this review article, the size and number of PUB subfamilies in 17 green plants are listed and compared along with those of yeast and humans, demonstrating the large expansion of this family in plant genomes. To illustrate the functions of PUB-mediated protein ubiquitylation and degradation, the authors comprehensively discussed the activities of over 50 PUB E3 ligases across many flowering plants, including Arabidopsis, rice, *Brassica rapa*, potato, tomato, cotton, *Medicago truncatula*, grape, tobacco, pepper, populus, pear, apple, wheat, and soybean. Given the activities of these known PUB E3 ligases, the authors categorized their negative or positive roles into abiotic stress responses, biotic stress defenses, and plant growth and development [13]. Notably, the roles of several *PUB* genes in pathogen defense, such as *A. thaliana* (*At*) *PUB12/13*, *AtPUB25/26*, and *OsSPL11*, were also discussed in the review by Chen et al. [14], highlighting the defensive role of PUB E3 ligases in plants. Interestingly, a PUB E3 ligase named armadillo repeat-containing 1 (ARC1) is involved in the sporophytic self-incompatibility commonly present in Brassicaceae [23,24]. However, the detailed biochemical pathway of ARC1 is yet unknown. Whether ARC1 shares some molecular mechanisms with pathogen defense PUBs for preventing self-pollination would be an interesting topic for further investigation.

Adopting a biochemical and molecular mechanistic perspective, Yu and Hua discussed the quality control of the 26S proteasome primarily found in yeast and human cells [18]. Although selective autophagy-mediated proteasome turnover was first discovered in Arabidopsis [25], several advanced biophysical studies on the intracellular dynamics of proteasomes were carried out in yeast and human cells. Based on these discoveries, Yu and Hua proposed four different activity statuses of proteasomes in cells: (1) reactive liquid–liquid phase separation (LLPS) droplets, encompassing an effective UPS pathway that includes active and functioning 26S proteasomes; (2) proteasome storage granules (PSGs), which protect dissociated regulatory particles and the core protease subcomplexes of proteasomes from being degraded; (3) soluble juxtanuclear quality control (JUNQ) aggresome-like structures of inactive proteasomes, which are degraded by other active proteasomes; and (4) insoluble protein deposit (IPOD) compartments, containing denatured and aggregated proteasomes for autophagy degradation. Future studies on this topic could investigate the presence and dynamics of these four statuses of proteasomes at the single-cell level. Advanced single-cell proteomics and cellular imaging may provide new insights into this topic. In addition to autophagy-mediated proteasome degradation, this review article also proposed a reciprocal degradation model by highlighting a list of Ub E3 ligases targeting key autophagy members for both proteolytic and non-proteolytic ubiquitylation processes [18].

The work by Retzer et al. [17] and Radjacommare et al. [16] characterized the function of RGLG1/2 ligases and a DUB protein, OTU5, respectively. Their discoveries are presented in the two research articles included in this Special Issue. In Arabidopsis, five *RGLG* genes are encoded. Previous work indicated a role of RGLG1/2 in Lysine-63 (K63)-linked polyubiquitylation and the steady-state levels of the auxin transport protein PIN-FORMED 2 (PIN2) [26]. To further comprehend the mechanism of RGLG1/2 in the endosomal trafficking of plasma membrane proteins, Retzer et al. utilized a cellular biological method in combination with mutant assays to demonstrate that RGLG1/2 themselves are localized in endocytic vesicles, for which their N-terminal protein myristylation motif is required. Through a complementation assay, the authors suggested that the localization of RGLG1 in endosomal sorting vesicles is essential for proper Arabidopsis development. However, the significance of RGLG1/2 in plasma membrane protein sorting was only evidenced by uncovering a moderate effect on the sorting and degradation of Ub-tagged PIN2 proteins that were associated with membranes. The authors demonstrated that the ratio of plasma-localized PIN2-Ub-VEN proteins to those in the cytosol increased significantly upon the elimination of RGLG1 and RGLG2 in *rglg1 rglg2* double mutants. These changes are dependent on ubiquitylation because blocking PIN2 ubiquitylation in the pin2^12K-R^ resulted in the retention of pin2^12K-R^-VEN on the plasma membrane regardless of the activity of RGLG1 and RGLG2. Given these data, the authors suggested that RGLG1 and RGLG2 impacted the sorting of internalized, ubiquitylated plasma membrane proteins. However, how and when these two mono-subunit RING finger E3 ligases target PIN2 for ubiquitylation remain elusive. In addition, there are likely other E3 ligases involved in PIN2 trafficking. Conversely, RGLG1 and RGLG2 might also target additional plasma membrane proteins for ubiquitylation. Further studies on this topic would shed light on the molecular mechanism behind the endosomal sorting of plasma membrane cargo.

Radjacommare et al. presented a novel function of a DUB protein, OTU5, in the suppression of flowering that is effected by influencing histone marks on the loci of *Flowering Locus C* (*FLC*) and *MADS Affecting Flowering* (*MAF*) *4* and *5*. Through a comprehensive study that includes expression analysis, quantitative Chromatin Immuno-Precipitation (qChIP), subcellular fractionation, the cellular localization of Green Fluorescence Protein (GFP)-fused OTU5, and Micrococcal Nuclease (MNase) treatment, the authors suggested that OTU5 is associated with chromatins, particularly in the loci of *FLC*, *MAF4*, and *5*, in the nucleus. Since the T-DNA insertion null mutant *otu5-1* has a pleotropic developmental phenotype, including early flowering, that mimics mutants harboring defects in subunits of the SWI2/SNF2-Related 1 Chromatin-Remodeling Complex (SWR1-C), the authors studied the genetic interaction between OTU5 and a SWR1-C subunit, ACTIN-RELATED PROTEIN 6 (ARP6). In the double knockout mutant *otu5-1 arp6-1*, the authors found a synergistic developmental phenotype, including leaf size, inflorescence structure, floral size, silique length, and root hair development. However, the deposition of H2A.Z on the loci of *FLC* and *MAF4* and *5* remained unaltered upon deletion of OTU5 but decreased if ARP6 was eliminated, leading the authors to conclude that OTU5 functions independently of SWR1-C. Using a similar genetic interaction study, the authors further demonstrated that the function of OTU5 is also independent of another epigenetic regulatory factor, HISTONE MONO-UBIQUITINATION 1 (HUB1). However, it is partially required for suppressing FLC-mediated flowering in autonomous mutants and FRIGIDA-expressing Col-0 plants. As the authors suggested in the article, the isolation of OTU5-interacting proteins and in vivo substrates would be essential to further illustrate the biochemical and molecular mechanisms of OTU5 in the regulation of flowering.

The remaining half of the articles in this collection studied the Ub E3 ligases involved in specific biological functions. The large expansion of the UPS has been recognized to contribute to the sessile lifestyle of plants for defending inevitable stresses. Accordingly, three review articles summarized the role of the UPS in stress response.

Nitric oxide (NO) is an unstable gas that can react with cysteine, leading to its oxidation or S-nitrosylation depending on whether the cysteine residue is released at the N-terminus or resides within a protein, respectively. It can also modify internal tyrosine residues, which is known as tyrosine nitration. Pande et al. [12] explained that N-terminal cysteine oxidation promotes the degradation of a substrate by the 26S proteasome via an N-degron pathway, which was exemplified by the Ethylene Response Factor VII (ERF-VII) family transcription factors. The NO-promoted degradation of ERF-VII is recognized as a NO sensor in plants. S-nitrosylation attacks internal cysteines either promoting or inhibiting the polyubiquitylation of a substrate, thus enhancing or suppressing its degradation by the 26S proteasome, respectively. However, tyrosine nitration has been found to promote ubiquitylation-mediated protein degradation. The authors listed six substrates whose ubiquitylation-mediated degradation is modulated by NO-mediated cysteine or tyrosine modifications.

Abscisic acid (ABA) is a master stress hormone. It is well known that protein ubiquitylation plays a major role in hormone-signaling transduction. Previous studies have reviewed a collection of ubiquitin E3 ligases involved in ABA biosynthesis, perception, and response [19,27]. In this issue, Coego et al. [11] focused on ABA perception by addressing how PYR/PYL/RCAR (for pyrabactin resistance/PYR1-like/regulatory components of ABA receptor) ABA receptors and PP2C (for protein phosphatase 2C) co-receptors are targeted by different ubiquitin E3 ligases. Interestingly, PUB22/23 and RGLG1/5 E3 ligases are also involved in ABA perception by targeting clade A PP2C proteins for ubiquitylation and degradation. Since ABA plays a vital role in plant drought tolerance, the authors proposed improving plant drought resistance by manipulating the ubiquitylation pathways targeting these two groups of proteins.

Chen et al. summarized the role of protein ubiquitylation in the fine tuning of the plant immune response [14]. This review article discussed the mechanisms whereby the stability changes of plasma-membrane-resident pattern recognition receptors (PRRs) and intracellular nucleotide-binding domain leucine-rich repeat receptors (NLRs) activate PAMP-triggered immunity (PTI) and effector-triggered immunity (ETI), respectively. The readers will benefit from examining the two figures presented in this article, which conceptually illustrated the regulatory role of protein ubiquitylation in these two pathogen responsive pathways. Notably, the authors also summarized the interplay between protein ubiquitylation and other types of PTMs, particularly the details of how phosphorylation regulates the activities of Ub E3 ligases. As the authors note, the further identification and functional characterization of novel Ub E3 ligases will advance this field. New technologies and approaches applied in ubiquitylome studies will certainly promote the progress.

The last article by Hand and Shabek summarized the present understanding of the role of ubiquitin E3 ligases in chloroplast function [15]. These E3 ligases include the suppressor of plastid protein import 1 locus 1 (SP1), Constitutive Photomorphogenesis 1 (COP1), PUB4, the Carboxyl Terminus of the HSP70-Interacting Proteins (CHIP), and Thermo-Tolerance 3.1 (TT3.1). The authors sequentially discussed the biological role and biochemical mechanisms of the ubiquitylation process mediated by each enzyme. As not only outer envelope membrane proteins but also intra-chloroplast proteins were recently found to be ubiquitylated, the authors proposed that the functions of a ubiquitin-dependent segregase, CDC48 (for cell division control protein 48), under a myriad of stresses warranted better understanding.

In summary, we appreciate the authors’ contributions to disseminating the importance of protein ubiquitylation in plant growth and development through this Special Issue of the *International Journal of Molecular Sciences*. It is wonderful to witness the depth and breadth of this work in the field of plant research. However, compared to the same research topics studied in yeast and humans, the scope of the plant protein ubiquitylation research society seems disproportional to the scale of the UPS in plant genomes. Although this may reflect a common issue in plant molecular biology research, exerting greater efforts toward training the next generation of young plant biologists would help unravel the mysteries of the plant UPS via means ranging from the fine tuning of individual ubiquitylation pathways to proteome-wide analyses. Clearly, the latter is not presented in this issue. The versatile role of protein ubiquitylation in plants is worthy of scrutiny not only with respect to helping develop elite crop varieties but also in terms of understanding fundamental biological questions.
